# Review and Perspectives on the Sustainability of Organic Aerogels

**DOI:** 10.1021/acssuschemeng.4c09747

**Published:** 2025-04-25

**Authors:** Carlos A. García-González, María Blanco-Vales, Joana Barros, Antonella Caterina Boccia, Tatiana Budtova, Luisa Durães, Can Erkey, Marta Gallo, Petra Herman, József Kalmár, Ana Iglesias-Mejuto, Wim J. Malfait, Shanyu Zhao, Lara Manzocco, Stella Plazzotta, Stoja Milovanovic, Monica Neagu, Loredana E. Nita, Patrina Paraskevopoulou, Anna Roig, Rosana Simón-Vázquez, Irina Smirnova, Željko Tomović, Clara López-Iglesias

**Affiliations:** † AerogelsLab, I+D Farma Group (GI-1645), Department of Pharmacology, Pharmacy and Pharmaceutical Technology, Faculty of Pharmacy, iMATUS and Health Research Institute of Santiago de Compostela (IDIS), 16780 Universidade de Santiago de Compostela, E-15782 Santiago de Compostela, Spain; ‡ Instituto de Investigação e Inovação em Saúde da Universidade do Porto, Rua Alfredo Allen 208, 4200-125 Porto, Portugal; § 9327 Istituto di Scienze e Tecnologie Chimiche (SCITEC) “Giulio Natta”, C.N.R., Via Alfonso Corti 12, 20133 Milano, Italy; ∥ Mines Paris, PSL University, Center for Materials Forming (CEMEF), UMR CNRS 7635, CS 10207, 06904 Sophia Antipolis, France; ⊥ 37829 University of Coimbra, CERES, Department of Chemical Engineering, Rua Sílvio Lima, 3030-790 Coimbra, Portugal; # Department of Chemical and Biological Engineering, 52979 Koç University, 34450 Sariyer, Istanbul, Turkey; ¶ Dipartimento di Scienza Applicata e Tecnologia, 19032 Politecnico di Torino, Corso Duca degli Abruzzi 24, 10129 Torino, Italy; ○ HUN-REN-DE Mechanisms of Complex Homogeneous and Heterogeneous Chemical Reactions Research Group, Department of Inorganic and Analytical Chemistry, 37599 University of Debrecen, Egyetem tér 1, Debrecen H-4032, Hungary; ∇ Laboratory for Building Energy Materials and Components, 28501 Swiss Federal Laboratories for Materials Science and Technology, Empa, Dübendorf 8600, Switzerland; ◆ Dipartimento di Scienze AgroAlimentari, Ambientali e Animali, 9316 Università di Udine, Via Sondrio 2/A, 33100 Udine, Italy; ☆ University of Belgrade, Faculty of Technology and Metallurgy, Department of Organic Chemical Technology, Karnegijeva 4, 11 000 Belgrade, Serbia; ★ Immunology Department, Victor Babes National Institute of Pathology, 99-101 Spl Independentei, 050096 Bucharest, Romania; ⊡ Pathology Department, Colentina Clinical Hospital, 10 Sos Stefan cel Mare, 020125 Bucharest, Romania; ⬠ Doctoral School Faculty of Biology, University of Bucharest, 91-95 Spl Independentei, 050663 Bucharest, Romania; ⬢ “Petru Poni” Institute of Macromolecular Chemistry, Romanian Academy, 41A Grigore Ghica Voda Alley, 700487 Iasi, Romania; ⌀ Inorganic Chemistry Laboratory, Department of Chemistry, National and Kapodistrian University of Athens, 15771 Athens, Greece; ◑ Institute of Materials Science of Barcelona (ICMAB-CSIC), Campus of the UAB, 08193 Bellaterra, Spain; ▼ 16784 CINBIO, Universidade de Vigo, Inmunology Group, Vigo, Spain, Instituto de Investigación Sanitaria Galicia Sur (IIS Galicia Sur), SERGAS-UVIGO, 36310 Vigo, Pontevedra, Spain; ◁ Institute of Thermal Separation Processes, Hamburg University of Technology, Eißendorfer Straße 38, 21073 Hamburg, Germany; ⧨ Polymer Performance Materials Group, Department of Chemical Engineering and Chemistry, Eindhoven University of Technology, 5600 MB Eindhoven, The Netherlands

**Keywords:** bioaerogels, circular technologies, sustainable production, aerogel recycling, waste upcycling

## Abstract

Aerogels are exceptionally lightweight materials characterized by their high open porosity and remarkable specific surface area, currently used across a wide array of industrial sectors from construction to energy storage and have great potential for expanding their applicability and unlocking new market opportunities. Driven by global economic growth and an intensifying environmental crisis, there is a growing demand for engineering innovations that prioritize sustainability. Aerogels are well-positioned to support these sustainability efforts. Their unique properties make them ideal for energy-saving solutions, environmental remediation, and more efficient use of resources. As the demand for eco-conscious technologies rises, aerogels are poised to contribute significantly to the development of greener, more efficient products and processes across multiple industries. The sustainability of aerogel technology is crucial for the mid-to-long-term future, yet its current status has been scarcely reviewed in the literature. This Perspective explores and critically reviews significant advances on organic and hybrid aerogels in the current socioeconomic scenario, with selected case studies endorsing their contribution to the UN Sustainable Development Goals. It also identifies research gaps while proposing innovative strategies to enhance the sustainability of aerogel production through the application of circular economy principles. Key strategies discussed involve the fabrication of aerogels using eco-friendly sources, such as biopolymers derived from biorefinery processes or from waste materials. Additionally, this Perspective examines the development of methods for the reuse, recycling, and end-of-life management of aerogels, along with the implementation of more efficient processing routes. Ultimately, this work highlights the need for comprehensive assessments of aerogel sustainability through life cycle assessment (LCA) and evaluations of safety and toxicity. By addressing these critical aspects, the potential of aerogels to contribute to a more sustainable future appears highly favorable from both commercial and research perspectives, paving the way for a circular aerogel economy and providing a lasting impact to the society in which we live.

## Graphical abstract

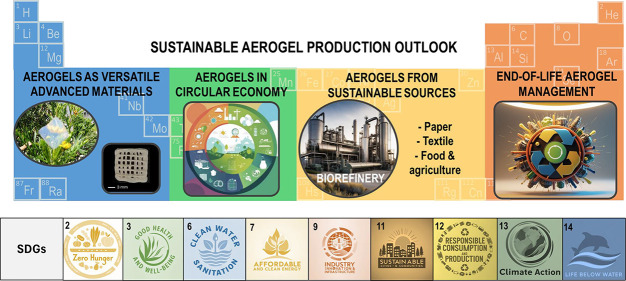

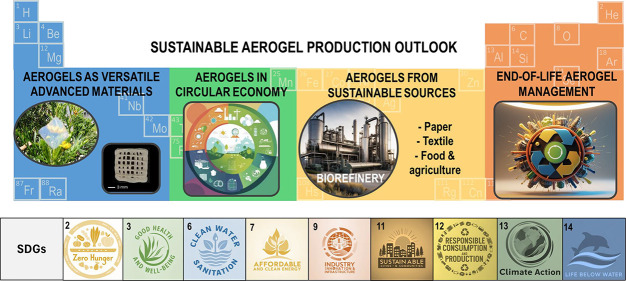

## 1 Introduction

New production and consumption paradigms are emerging worldwide due to an overall expense increase derived from the scarcity of raw materials and low-priced energy.^1^ Access to raw materials is of enormous importance for the economic stability of most countries, as they contribute to a robust industrial foundation, serve to produce daily goods, and are inextricably linked to the development of clean technologies.^2^ However, especially after the COVID-19 crisis, global economic growth has resulted in an industrial bloom that accentuated the shortage of some resources, increasing industrial supply periods and prices with consequent economic inflation. As a result, recent international policies are addressing the identification of critical raw materials (CRMs) for multiple industrial sectors in Europe, the US, and Japan.^2–6^ China is also of significant geopolitical importance, from mining and processing to the manufacture and trade of CRMs.^7^

The so-called *Twin Transition* envisions a carbon-neutral society by reinforcing digital breakthroughs and promoting green and sustainable technologies.^8^ The significant material and energy reliance of Europe on third countries fostered the implementation of strategic projects for economic recovery and transformation (*Next Generation EU*). This enables exploitation of technologies and increases prospects for energy- and cost-efficient resources and process innovations.^2^ Climate-neutral circular economy approaches are actively explored and implemented in all sectors and countries, with product reuse and recycling as well as waste upcycling. The European Commission (EC) adopted the *New Circular Economy Action Plan* in 2020 to reduce strain on natural resources, ensure sustainable growth, and meet the EU’s 2050 climate neutrality target.^9^ The driving forces are the protection of the environment, the reduction of raw material dependence, and the sustainable boost of economic growth (create jobs, increase competitiveness, stimulate innovation, increase service life of products, improve quality of life in the long term). Similar initiatives have been launched by the USA (Inflation Reduction Act),^10^ Japan (Green Innovation Fund),^11,12^ and China.^13^

These new regulations, as well as market and societal demands, require the development of sustainable, innovative, and advanced functional materials. A prominent class of materials that could address some of the mentioned challenges are aerogels, nanostructured materials endowed with unique properties, e.g., high specific surface area (usually above 100 m^2^/g), low bulk density (usually below 0.2 g/cm^3^), and open porosity (usually higher than 85% with a high presence of interconnected mesopores). Aerogels are especially attractive for a wide range of applications, from thermal insulation in buildings and industrial facilities^14^ to environmental (adsorbents for air, soils,^15^ and water remediation,^16,17^ selective binders for CRMs recovery,^18,19^ sensors and catalysts,^20,21^ sound absorbers^22^) and biomedical uses (scaffolds for regenerative medicine,^23^ thermal insulators for photothermal oncotherapy,^24^ dressings for wound healing,^25^ drug carriers^26^). In addition, aerogels can be found in emerging applications in the food sector, where they may act as packaging materials with advanced functionalities (e.g., cushioning effect, thermal insulation, release or absorption of desired/undesired compounds) or food ingredients (e.g., fat replacers, delivery systems for active compounds, etc.^27–29^ ). The superior properties of aerogels in different fields resulted in a high scientific impact, such that the prestigious authority IUPAC (International Union of Pure and Applied Chemistry) identified aerogels as one of the Top Ten Emerging Technologies in Chemistry in December 2022.^30^

From an industrial point of view, the aerogel market is estimated at 1,155 million USD by 2025, with an average annual growth rate of 26% until 2030.^31^ Advances in recent years encompass the use of various sources (inorganic materials, organic synthetic polymers, natural polymers, carbons, hybrid materials) for single component or composite aerogels, diverse morphologies (powder, beads, monoliths, mats, boards, films), and dimensions (from nanometers to meters), along with modeling, production scale-up, and health and safety assessments.

From a sustainability point of view, aerogel producers should decide on an energy-efficient drying process, probably the most critical step of the production line, with consideration on the material source and the intended use and performance. Furthermore, the rational use of resources should include minimization of materials use, reuse of solvents, and recycling of unspent precursors, toward zero-waste in the production line. These also must be contemplated for the pretreatment of raw materials (e.g., extraction, derivatization, milling, purification) and the postprocessing of aerogels (e.g., polishing, cutting, milling, carbonization, sterilization). Production costs can be optimized through the reduction of cycle time (for batch production), the increase of throughput (for continuous production), and the integration of processes. Studies on most of these technological aspects have been recently reviewed in the literature,^32,33^ but life cycle and sustainability assessment studies are still scarce.^34,35^

While minimizing aerogel manufacturing time and costs, business strategies should also focus on sustaining and further extending the niche markets for this material. This Perspective also addresses the circularity of different wastes that may feed the production of aerogels with an increased circular material use rate, reduced usage of raw materials, and lower energy consumption. For instance, developing more sustainable thermal insulation products could have important environmental and economic impacts as the building thermal insulation market is projected to almost double in the next ten years (from 25 billion USD in 2022 to 45 billion USD in 2032 ^36^). Similarly, the introduction of circular economy principles by using waste products in the wound healing domain will have an enormous economic impact, as the global wound care market is expected to expand to 28 billion USD by 2029. Also, aerogels intended as food grade oil structuring ingredients will position in the market of fat replacers, which reached 2.2 billion euros in 2022 in Europe and with an annual growth rate of ca. 6%.

The societal impact will be tied to both the lowering of the countries’ consumption footprint and their raw material self-sufficiency. However, sustainability and end-of-life management of aerogels, as well as rational use of resources for their production, have received little attention thus far, providing a technological and commercial challenge to the current state-of-the-art. Sustainable aerogel production has recently evolved, with significant and exponential research growth rates.^37^ The expected roadmap for this topic from different perspectives and approaches will be discussed in the following sections of this Perspective: (i) identifying aerogel positioning in the United Nations (UN) Sustainability Development Goals (SDG) context (Section 2); (ii) performing a critical review of significant advances and searching for research gaps in innovative and (still) underexplored use of sustainable sources for aerogel production from biorefineries (Section 3), (iii) addressing wastes and byproducts (Section 4); (iv) proposing various options for end-of-life of aerogel wastes by their recycling, reprocessing or upcycling (Section 5); (v) proposing the implementation of process integration strategies and emerging technologies in aerogel production to minimize the consumption of resources and decrease energy use (Section 6). Finally, LCA considerations of organic aerogels (Section 7), end remarks and other remaining challenges in the sustainable production of aerogels are compiled and emphasized (Section 8).

## 2 Aerogels in the UN SDGs context

The UN established 17 SDGs as part of the 2030 Agenda for Sustainable Development. To meet their requirements, the economic paradigm must change from linear to circular. This shift emphasizes responsible material usage and disposal, as well as recycling. Recycling has several advantages, including energy savings, natural resource preservation, and waste reduction. The management of solid waste has a significant impact on community health, as well as the natural environment.^38^ Governments assign 4–10% of their total budgets to this duty as a first attempt to fulfill SDG13 (climate action).

Biobased aerogels (or bioaerogels) obtained from natural polymers are advantageous for their abundance, biocompatibility, and biodegradability.^28,29^ Bioaerogels are in harmony with the SDGs due to their inherent degradability, which ensures their disposal in accordance with the circular economy. As many bioaerogels are biocompatible, it opens the sustainable application of these solutions in the biomedical, environmental, food, and packaging fields, aligning with SDG3 (good health and well-being) (Figure 1). From one side, these eco-friendly solutions are sustainable alternatives to standard nonrenewable medical or packaging materials, which reduce environmental impact directly throughout their life cycle. On the other hand, biocompatibility makes biobased aerogels food-grade materials, supporting their use as ingredients for sustainable and healthier diets. These sustainable applications of aerogels are being studied using different renewable sources such as chitosan, cellulose, poly­(lactic acid), proteins, etc.^39,40^ These polymers could be obtained by the valorization of agricultural wastes or food industry byproducts (e.g., sugar cane or crustacean shell),^41^ thus fulfilling SDG12 (responsible consumption and production) and SDG2 (food security, improved nutrition, and promotion of sustainable agriculture). Additionally, aerogel sorbents manufactured by the valorization of agricultural and food wastes, or aerogels obtained from mineral phases (e.g., silica and clay), are sustainable alternatives with a high capacity for water remediation and recovery of CRMs, such as Pt, Pd, Ag, Au, lanthanides, or actinides from aqueous media.^42–44^ In this regard, aerogels may contribute to SDG6 (clean water and sanitation) as well as to SDG14 (life below water).

**Figure 1:**
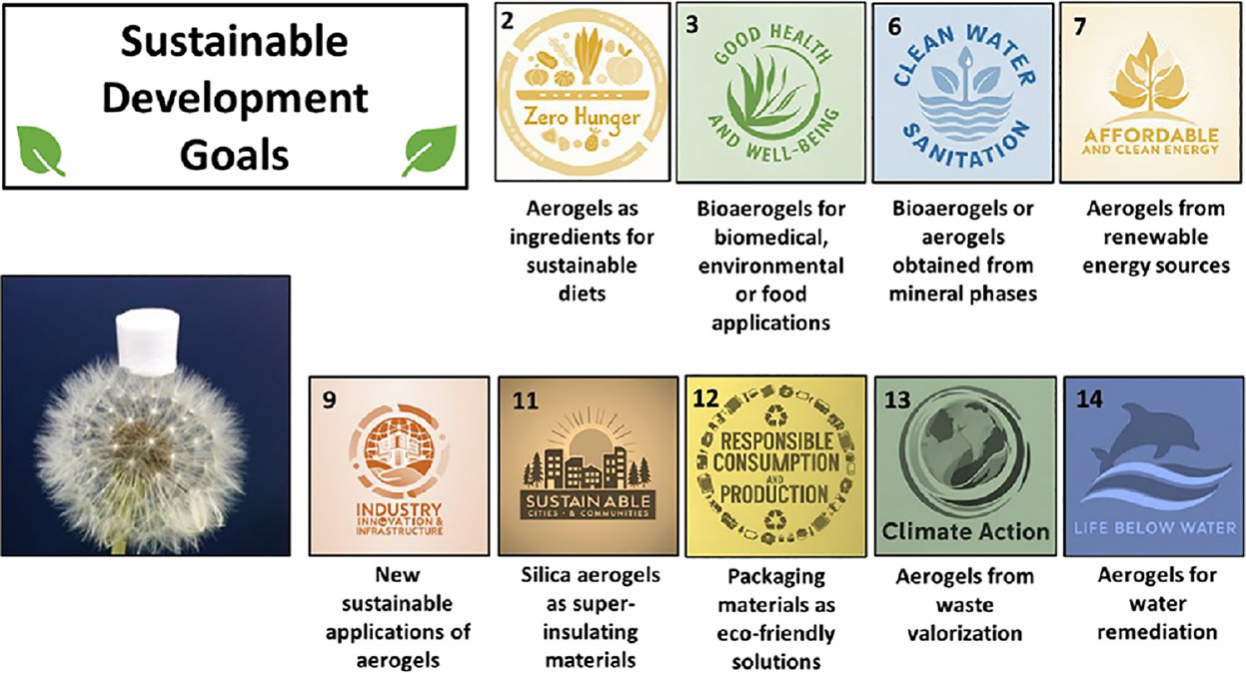
Outlook of the most remarkable contributions of aerogels to the UN sustainable development goals.

Buildings and their facilities must have minimum energy requirements since the publication of the *2010/31/EU Energy Performance Building Directive* by the EU. Aerogels represent a new generation of thermal insulation materials with remarkable engineering applications due to their lightweight and extremely low thermal conductivity, as their nanostructure restricts the conductive heat transfer and the movement of gas molecules. Research and development on aerogels as new superinsulating materials is a way to commit to this policy with the ultimate goal of designing net-zero energy buildings. All these efforts are well linked with SDG11 (sustainable cities and communities) (Figure 1).^45^ As an example, silica aerogels are extremely lightweight and highly effective thermal insulators with a thermal conductivity significantly lower than other commercial insulating solutions. This advantageous skeleton has fostered their use in green building construction or clothing, energy production, and automotive, aerospace, and military industries, thus being in line with SDG7 (affordable and clean energy) (Figure 1).^46,47^ Other applications of silica aerogels are being explored like their use as acoustic absorbers due to the low velocity of the sound transmission throughout the matrix of the aerogel thereby promoting SDG9 (industry, innovation and infrastructure).^48^ In these applications, research should focus on improving the mechanical, processability, and stability properties through the use of organic (polyurethane^49^ or polyimide^50^), carbon,^51^ or hybrid (silica-organic^52^) aerogels, as they remain as the biggest challenges for aerogel efficiency and long-term performance.^53,54^

## 3 Aerogels from a Biorefinery Approach

Alternative sustainable sources for aerogel production are currently under evaluation. A key selection factor is the abundance of the source at an affordable cost to secure the supply. The biorefinery as a source of raw materials is especially attractive from an environmental standpoint (Figure 2). Indeed, biorefinery technologies allow for the efficient valorization, fractionation, and transformation of different biomass feedstocks in terms of mass and energy consumption.^55^ Several economic and life cycle assessments available elsewhere strongly support the implementation of this biorefinery concept.^56–58^ Conversely, this section focuses on the identification of research gaps.

**Figure 2:**
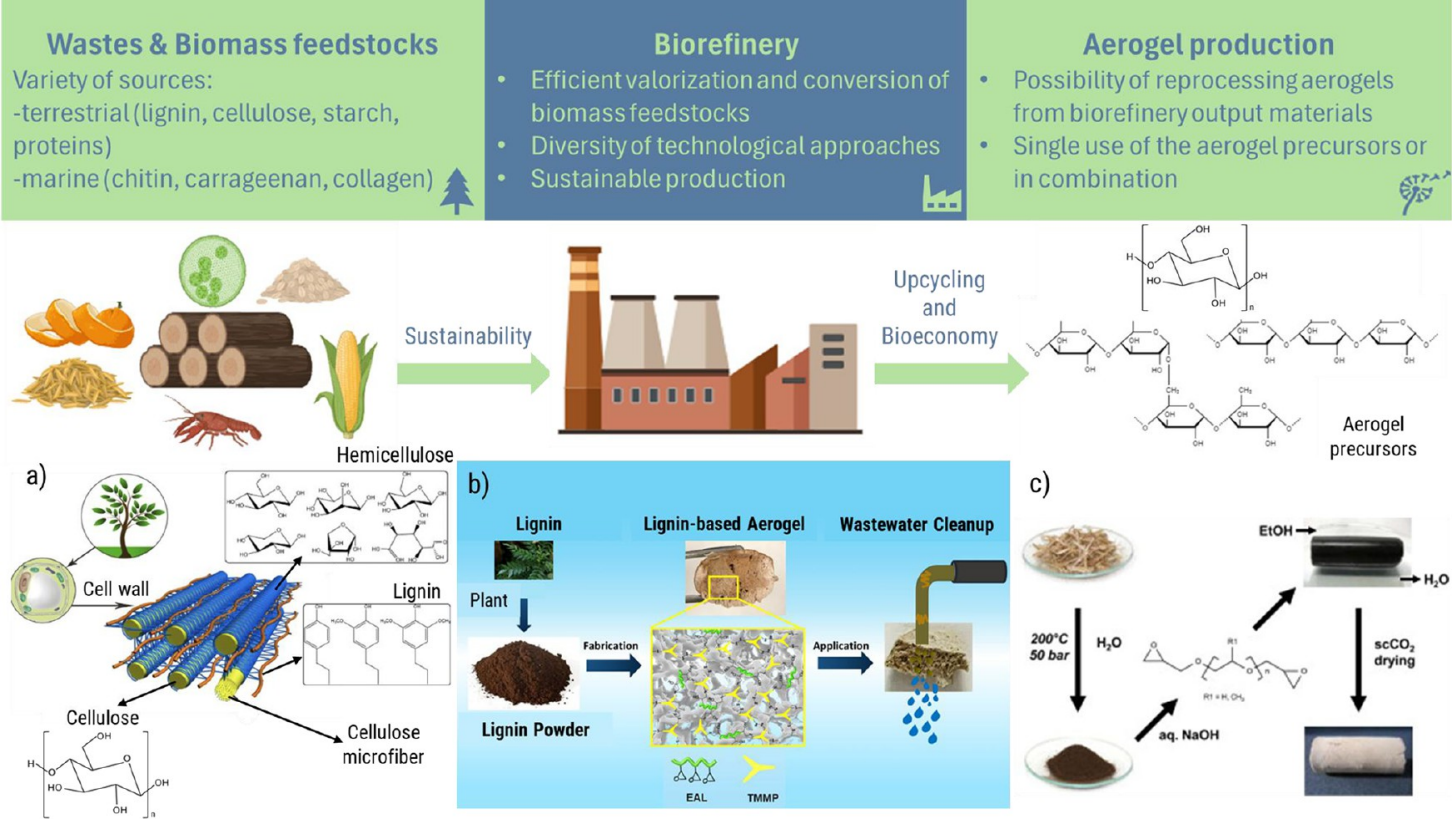
Implementation of biorefinery approaches in aerogel production: (a) Lignocellulosic biomass chemical composition.^55^ Adapted with permission from ref 59. Copyright 2017, Elsevier. (b) Lignin-based aerogel for wastewater remediation.^60^ Adapted with permission from ref 60. Copyright 2022, Elsevier. (c) Aerogel preparation from lignin derived from wheat straw.^61^ Reprinted with permission from ref 61. Copyright 2014, Elsevier.

Biorefineries employ several technological approaches, the most important of which depend on biomass feedstock attributes (e.g., origin and amount of residues) and product standards targets (yield, purity). In the biobased economy paradigm, using these biorefinery output materials to make aerogels can contribute to upcycling into high added-value advanced materials. The sustainable production of materials from biomass should be supported not only by the source itself but also by using eco-friendly and safe production technologies with a low CO_2_ footprint, avoiding the use of hazardous reagents, and preventing mass losses during the process cycle. Aerogel end-of-life and waste management should also be considered (cf. Section 5), as they should be safe for producers and customers, sustainable for the ecosphere, and economically feasible. The management of aerogel leftovers after use should be defined by design; otherwise, the sustainable production approach will be diluted or neglected.

Two main biorefinery sources can be used to produce aerogels (bioaerogels): terrestrial and marine tissue wastes. The most common raw materials from terrestrial vegetal wastes are lignin, cellulose, pectin, and starch, while silk fibroin, gelatin, whey protein, and keratin are from terrestrial animal wastes and byproducts.^62–69^ Agarose, chitin and derivatives (chitosan), carrageenan, and collagen and derivatives (gelatin) are the most used raw materials from marine wastes.^62,67,70,71^ These raw materials are used alone or in combination with natural or synthetic components, for example, cross-linkers or other admixtures (e.g., nanoparticles), to tune the physicochemical properties of aerogels. It should be noted that the use of biorefinery-derived aerogels may be limited in some areas, such as in life science applications (biomedicine, pharmaceutics), due to the type, quality, and variability of the biorefinery source.^26,27^ Finally, the choice of additives for the material design should be rational to avoid the underscoring of aerogel sustainable production.

## 4 Aerogels as a Green Way to Waste Upcycling

Economic development, population growth, and fast urbanization are associated with a significant increase in consumption and a consequent increase in waste. Currently, 2.1–2.3 billion tons of only municipal solid wastes per year are generated worldwide^72^ and the global circular material use rate is generally low (e.g., only 11.5% in 2022 in EU-27).^73^ This translates not only in high managing and disposal costs but also in wasting of resources (land, water, and energy) necessary to produce goods.

Waste recycling refers to the reuse of existing waste material, which frequently results in lower-quality products with limited applications. The concept of waste upcycling refers to its reuse to fabricate upgraded or added-value materials, also known as waste valorization.^64^ Aside from the transformation of the waste into relatively pure raw materials via biorefinery processes (cf. Section 3), waste upcycling can also be accomplished by simpler processing involving the conversion of the integral biomass into valuable derivatives.^74,75^ Because no strong purification or extraction is performed in this situation, the high value of the resulting materials can only be achieved through complex supra- or macromolecular structures or architectures.^76,77^ As previously mentioned, aerogels are regarded as high-value materials with outstanding properties. However, applications such as food and beverage (direct) packaging, cosmetics, or technical clothing are usually not considered for waste-derived aerogels,^78^ due to a general lower purity than those obtained through biorefinery.

A first important step to allow for the usage of integral biomass as aerogel precursor would be waste upgrade from waste to byproduct. According to the waste framework Directive (Directive 2008/98/CE), a waste ceases to be a waste and is classified as a byproduct if specific conditions are met. For instance, the conversion of wasted biomass into food-grade aerogels would necessitate the establishment of a dedicated production chain, commencing with waste collection. The latter should adhere to rigorous food regulations to ensure the minimization of safety risks. In this context, it should also be pointed out that, in most cases, biomass waste produced by food industries or consumers is currently classified as a byproduct since it is utilized in biogas production, composting, or animal feed.^79^ However, these strategies result in products of diminished value in comparison to those reaching the market. In contrast, the use of food byproducts as aerogel precursors would allow their upcycling, leading to high value materials.

Nguyen and co-workers conducted a thorough analysis of aerogels produced from waste materials,^38^ focusing on the main outcomes of the research, such as aerogel characterization and performance. Conversely, in this section, an overview of the process will be provided, from the collection and processing method of the integral biomass into aerogels to their final performance and applicability of the resulting aerogels. The environmental impact of the whole process, from waste production to final reuse or disposal, will be discussed, and research gaps and future directions will be identified.

### 4.1 Waste-Based or Byproduct-Based Aerogel Preparation

Aerogels can be produced from waste or byproducts that are dissolved or dispersed in a suitable medium, after chemical or physical treatment that will promote the formation of a 3D network. The mixture of textile fibers waste with a standard silica sol is an option to obtain composite aerogels with improved thermal and acoustic insulation properties, high water-contact angle, and high flexibility.^80^ Another example is the dissolution of recycled tire granulate rubber by an oxidant acid to form a rubber sol that can be easily mixed with the silica sol before its gelation. In this way, a hydrophobic efficient thermal insulator can be prepared, formed by a continuous rubber-silica aerogel matrix.^81^ It can also be extended to compound other inorganic or organic sol–gel-derived phases. In some cases, nonsolvent induced phase separation and formation of a network, as for example for aerogels prepared from cellulose-based textile.^82,83^ Alternatively, waste-derived aerogels can be produced by directly mixing food waste or byproduct materials with EtOH or water/EtOH mixtures with increasing concentrations of EtOH^84^ followed by supercritical drying. In this way, water originally present in the waste material is substituted with ethanol, which is then removed via supercritical drying. This process is particularly convenient when the waste material already presents a natural architecture (e.g., plant wastes and byproducts) suitable for aerogel production.

### 4.2 Waste Streams

#### 4.2.1 Food and Agriculture Waste

According to the FAO 2024 “Global Facts”,^85^ around 8–10% of global greenhouse gas emissions are associated with food that is not consumed. It was calculated that ca. 931 million tons of food were lost in the supply chain, from after harvest, and prior to reaching retail shelves in 2021. Meanwhile, 1.05 billion tons of food was wasted in households, food services, and retail in 2022. This represents ca. 30% of all food produced worldwide for human consumption raising significant ethical concerns.

Food byproducts are often represented by animal or plant tissues, which are discarded during food processing such as swarfs, substandard materials, or exhausted matrices, e.g., from fruit juice and oil extraction. These cellular tissues can be treated as gel-like materials made by a complex biopolymer network, mainly structured by cell wall cellulose fibers, embedding water within intra- and intercellular spaces. The direct conversion of these “gel-like” materials into “aerogel-like” ones could thus represent a possible approach to sustainably turn critical biomass into shelf-stable ingredients without further generation of waste. For instance, byproducts (external leaves and core) from industrial fresh-cut processing of iceberg salad were submitted to water-to-ethanol substitution and supercritical CO_2_ drying, producing a white aerogel-like material which could be used as packaging, absorbent, or an innovative carrier for both lipophilic and hydrophilic compounds.^84^ When the same procedure was applied to homogenized substandard peas, colorless powders without vegetable sensory notes, but with high nutritional value and technological functionality, were obtained.^86^

Food byproducts have also been used to produce fully biodegradable pure^87^ and hybrid^88^ aerogels with high porosity. In the latter case, the food residue was homogenized and structured into a superimposed architecture by means of additional gelling agents (e.g., k-carrageenan, poly­(vinyl alcohol)).^88^ This approach allowed turning discarded salad leaves into aerogels for food applications. An important limitation of waste-derived natural polymers resides in their high hydrophilicity, which has derived into strategies for hydrophobic enhancement of the resulting aerogels.^89^ Lignocellulosic aerogels were produced from spent ground coffee and apple pomace^90^ with enhanced hydrophobicity through silanization in a liquid phase or by vapor deposition. Silanes are common hydrophobizing agents for cellulose, forming polysiloxane structures by reacting with the hydroxyl groups of the cellulose fibers through a condensation reaction.^91^ However, it should be noted that Si–O–C bonds are easily hydrolyzable in the presence of water.

#### 4.2.2 Textile Waste

Besides recent awareness about the use of fast fashion,^92^ reality shows an increased consumption of textile products from 78 to 103 million tons in the past decade, and this tendency is still growing. Each European citizen discards an average of ca. 11 kg of textiles annually, most of which are disposed in landfills or incinerated.^93^ Landfill disposal has been forbidden in the European Union since 2016; incineration leads to the release of harmful chemicals and produces significant CO_2_ emissions. The textile recycling process possesses a poor economic value, so research groups have explored engineering solutions to provide added value, such as using the fibers for mechanical reinforcement of aerogels.^80^ In the case of cotton fibers, they can also provide a buffer effect for humidity regulation.

About 30% of textiles are based on cellulose (cotton, viscose, Tencel). Until now, the main option of upcycling cellulose-based waste textile has been dissolving the fabric and spinning fibers or casting films; this work is mainly performed on a laboratory scale or by small companies. Recently, it was demonstrated that it is possible to make aerogels from cellulose-based textile waste.^82,83,94^ Cellulose fabric, cotton, or viscose was dissolved in ionic liquids, coagulated in water or in ethanol, and dried with supercritical CO_2_. The bulk density was from 0.07 to 0.2 g/cm^3^, and the specific surface area was from 300 to 400 m^2^/g. These aerogels could be produced in the shape of monoliths or particles (Figure 3).

**Figure 3:**
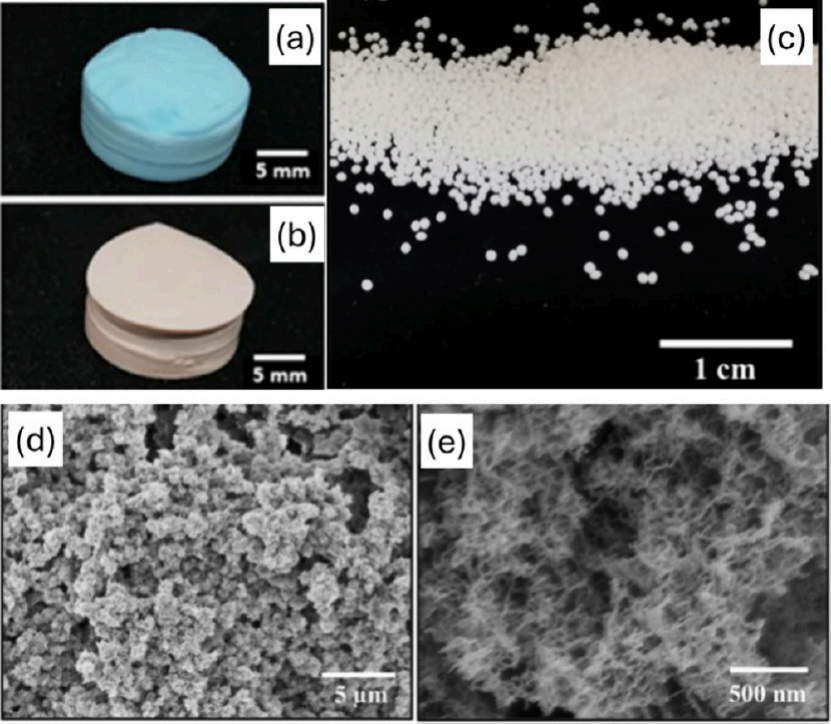
Aerogels prepared from waste textile ((a) rayon, (b, c) viscose) in the shape of monoliths^82^ (bulk density 0.1 g/cm^3^; specific surface area 330 m^2^/g; porosity >90%) and beads^83^ (roundness 0.97–0.98, density 0.08 g/cm^3^; specific surface area 400 m^2^/g; porosity 97%); (d, e) their internal morphology, at different scales and similar for all aerogels, is imaged by scanning electron microscopy.^82,83^ Adapted with permission from ref 83. Copyright 2024, Springer Nature.

#### 4.2.3 Paper Waste

Paper is one of the most recycled municipal waste streams, and ca. 72% of paper and pulp are produced from recycled sources.^95^ However, the recycling process after several cycles causes fiber damage and reduced cellulose molecular weight.^96^ The life cycle of this low-quality paper waste could be extended by its transformation into new and valuable aerogel-based products. Paper waste was used as a carbon source for the fabrication of carbon-based aerogels with outstanding performances as absorbents from water, with sorption rate values at least 2 orders of magnitude higher than those of activated carbon.^97^ A typical preparation procedure consists of dispersing paper waste in water under stirring to obtain the pulp, drying of the fibers, and thermal treatment under an inert atmosphere (including pyrolysis and chemical vapor deposition). These materials achieved exceptional surface area values (up to ca. 900 m^2^/g), finding applications in the recovery of organic pollutants,^97^ antibiotics, and oils/organic solvents from water.^98^

#### 4.2.4 Plastic Waste

Around 400 million tons of plastic are produced annually worldwide.^99^ Most plastic waste management strategies include direct disposal in landfills/the sea and combustion, both of which have an important negative environmental impact. Polyethylene terephthalate (PET) is one of the most common plastics and is used in all sorts of consumer products due to its high stability and resistance to degradation.^100^ This results in great interest in developing biodegradable alternatives that are stable enough for packaging applications. Besides the development of sustainable alternative materials, recycling of existing PET plastics is a major concern due to poor economic value and lack of environmentally friendly PET processes. Recycled PET fibers and bottles were also used to produce aerogels of high-value engineering applications (e.g., thermal insulation, CO_2_ capture, and oil spill cleaning) but were only scarcely tested as PET cryogels^100,101^ and aerogels^197^ so far.

## 5 End-of-Life of Aerogel Waste Management by Its Recycling, Reprocessing, or Upcycling

Reuse, reprocessing, repurposing, and recycling are related concepts in the management of used materials, but they have distinct meanings. Reuse involves using an item again for the same or a similar purpose without significant modification. Reprocessing involves treating or processing materials to make them suitable for a new use. This often includes physical or chemical changes to return the material to a usable state. Repurposing is the act of using a product or material for a different purpose than it was originally intended, often with little or no alteration. Recycling involves converting waste materials into new materials or products, with the same application or not. All these concepts, especially the first three ones, are in many cases used indistinguishably, as very often reprocessing (also known as regeneration) is a prerequisite for reuse, which can also be considered as a similar, but more general, term to repurposing; recycling also requires reprocessing and/or implies reuse. The application of these concepts to aerogels up to now has been mainly devoted to recycling of the organic solvents used in the synthesis procedures or toward recycling of CO_2_ used for supercritical drying of gels. However, recycling, reuse, reprocessing, or repurposing of aerogels themselves has not been explored much yet.

The reuse or reprocessing of aerogels is an emerging research field for catalysis (i.e., reuse of aerogel catalysts) and environmental remediation (e.g., reuse of aerogel sorbents). It should be pointed out that, when it comes to materials used as catalysts or sorbents, sustainability can be assessed based on several factors, including efficiency, longevity, and potential for reuse or recycling. In the context of a circular economy, sorbent materials that can be reused without significant loss of capacity are more sustainable because they can be used over multiple cycles, reducing the need for continuous production and disposal of new adsorbent materials. Although there are only a few such examples compared to the total number of aerogels explored for these specific applications, this can be attributed to the novelty of the concept rather than to the materials being unsuitable for reuse. The number of publications reporting reprocessing/reuse studies has recently increased significantly. This trend will reveal even more aerogel materials that can be reprocessed/reused.

In the field of catalysis, monolithic metal-doped carbon aerogels provide an example of easily reusable catalysts.^102^ Carbon aerogels bearing metal nanoparticles dispersed homogeneously throughout their volume (Figure 4, M@C aerogels; M: Fe, Au, Pt, Pd, Ni, and Rh), prepared via pyrolysis of ferrocene-bearing polyamide aerogels and subsequent transmetalation, exhibited catalytic activity toward (a) oxidation of benzyl alcohol to benzaldehyde (Au@C or Pt@C); (b) reduction of nitrobenzene by hydrazine to aniline (Fe@C) and Heck coupling reactions of iodobenzene with styrene or butyl acrylate (Pd@C), with yields in the range of 85–98%. Due to their monolithic shape, all these catalysts could easily be removed from the reaction mixture. More importantly, these catalysts were reused five times just by transferring them into a new reaction mixture, without any need for reprocessing. The yields at the end of the fifth cycle were in the 70–86% range. Similarly, monolithic Cu@C aerogels, prepared via pyrolysis of Cu­(II)-chitin aerogels, have been proven to be efficient chemoselective catalysts for the selective reduction of maleimides to succinimides under mild conditions, using hydrosilane as a hydrogen source.^103^ Cu@C catalysts could be removed from the reaction mixture via filtration and then reused for at least six times without significant loss of activity.

**Figure 4:**
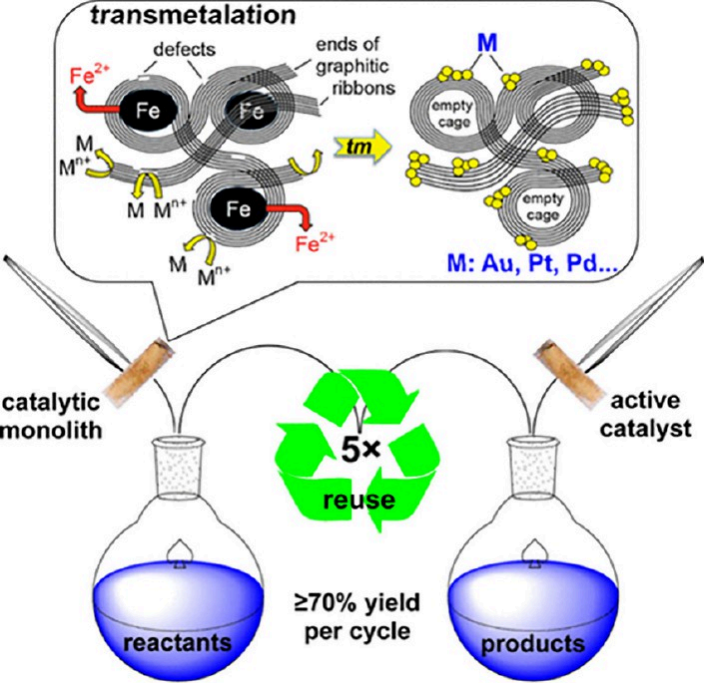
Transmetalation of Fe@C aerogels to M@C aerogels (M: Au, Pt, Pd, Ni, and Rh) leading to reusable catalysts. Adapted from ref 102. Copyright 2016, American Chemical Society. (The figure has been edited from its original version, where the authors had used the term “recycle”; in the context of the current understanding of the terminology, “recycle” is replaced with “reuse”).

In the field of environmental remediation, more examples of reusable aerogels can be found. For these uses, reprocessing is also needed as the materials after adsorption/absorption of pollutants need to be stripped from the pollutant and regenerated before they can be used again. As demonstrated by the representative examples presented below, the regeneration process can be simple or quite tedious, depending on the materials and the specific application.

Crystalline imine covalent–organic framework (COF) aerogels, prepared from the reaction of multifunctional amines with multifunctional aldehydes, were tested toward various environmental applications.^104^ Depending on their chemical structure and pore size, COF aerogels could be used for the efficient removal of a range of organic solvents (both miscible and immiscible with water), organic dyes, and inorganic micropollutants (gold nanoparticles) from water and for the capture and retention of iodine vapor. COF aerogels with the best sorption capacities (16–35 times their own weight) for organic solvents were solvent-exchanged with ethanol and dried again using supercritical CO_2_. The reprocessed aerogels exhibited practically no loss of crystallinity or sorption capacity for 10 cycles. Similarly, the ones that showed the best removal efficiency (97%) for methylene blue were washed with acetone and methanol, and they were reused for four more cycles with practically the same performance in the removal of the dye.

Biocompatible biopolymer-based aerogels are by design suitable for environmental applications, as they have various potential coordination sites (e.g., −COO^–^, −OH, −NH, −NH_2_). One such example is polyurea-cross-linked alginate (X-alginate) aerogels, a new class of materials recently introduced,^105–107^ which can be prepared from preformed alginate gels via reaction of the functional groups on the surface of the skeletal framework of the alginate (i.e., −OH) with multifunctional isocyanates, leading to the formation of a nanothin polyurea coating over the entire alginate framework, which enhances the mechanical strength of the materials. Use of different multifunctional isocyanates allows for tuning of the material properties from the chemical composition perspective.^108^ More specifically, X-alginate aerogels derived from tris­(4-isocyanatophenyl)­methane (TIPM) are extremely stable in diverse aqueous environments (no swelling, shrinkage, or disintegration has been observed), including seawater. These materials can efficiently uptake organic pollutants (solvents,^109^ organic dyes^110^) and inorganic pollutants, such as U­(VI),^19,111^ Pb­(II),^109^ Eu­(III),^112^ Th­(IV),^112^ Am­(III),^111^ and Hg­(II).^110^ Their properties, combined with their high sorption capacity, allow for the reuse of these materials for several cycles. For example, in the case of Pb­(II)^109^ and Hg­(II),^110^ the materials can be treated with an aqueous solution of Na_2_EDTA, washed with water, and reused for at least three times (Figure 5a) without significant loss of performance. In the case of U­(VI), which is the most extreme case, as X-alginate aerogels adsorb twice their mass (2 g g^–1^), the materials can be reused for at least five times.^19^ Similarly, silica–gelatin hybrid aerogels, prepared via cogelation of gelatin and tetramethoxysilane, have shown high selectivity for the adsorption of aqueous Hg­(II) in the presence of multiple competing ions, e.g., Cu­(II), Cd­(II), Co­(II), Pb­(II), Ni­(II), Ag­(I), and Zn­(II), and can be reused for five times after treatment with Na_2_EDTA.^113^

**Figure 5:**
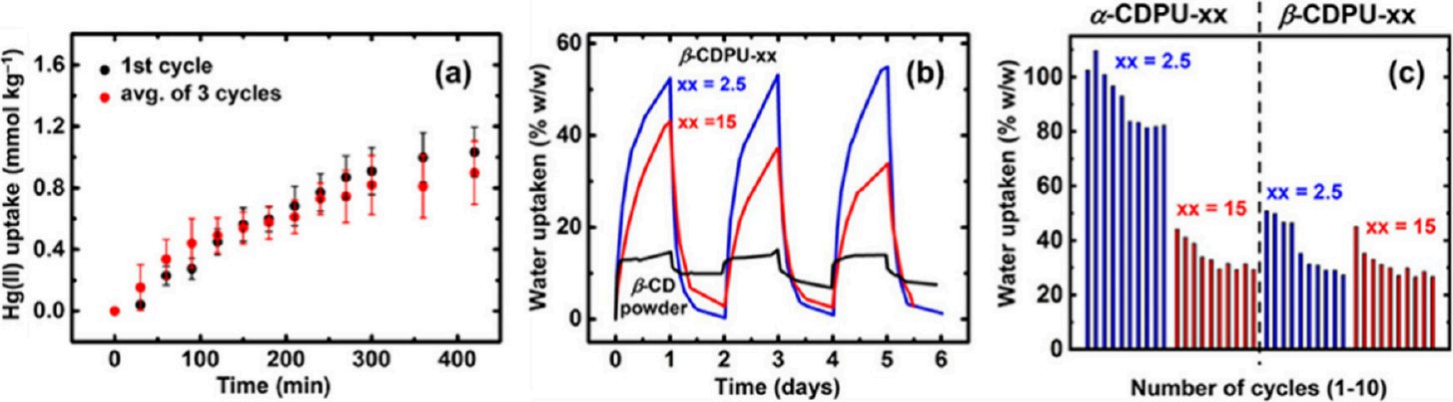
(a) Reusability of X-alginate aerogels for adsorption of Hg­(II) (*C*_initial_ = 100 ppb), after reprocessing that includes washing with an aqueous solution of Na_2_EDTA and water. (b) Three consecutive cycles of water vapor uptake between a high (99%) and a low (10%) relative humidity environment by β-CDPU-aerogels. (c) Ten consecutive cycles of water vapor uptake monitored every 24 h for α-CDPU- and β-CDPU-aerogels (xx: %w/w concentration of monomers in the sol). (b, c) Adapted from ref 114. Copyright 2019, American Chemical Society.

Another example of reusable aerogels with little treatment before being reused is α- or β-cyclodextrin-based polyurethane (CDPU) aerogels.^114^ Upon exposure to a high-humidity (99%) environment at room temperature, these aerogels showed high water vapor absorption capacities (up to 108% w/w). These materials outperformed by far the corresponding cyclodextrins (in powder form) and the commercial products silica gel and Drierite (absorption capacities up to 20% w/w) (Figure 5b,c). Most importantly, owing to the balance of enthalpic and entropic factors, absorbed water could be released by just reducing the relative humidity of the environment to 10% at room temperature. CDPU aerogels can be reused for 4–5 times without any significant loss in performance, and they can be reused for at least another 5–6 times, as the water vapor uptake seems to have been stabilized, operating at 80% of their maximum performance. This facile regeneration is rather rare and practical, and these materials could be used as desiccants in places where cold humid nights alternate with hot dry days.

Increasing interest in aerogel production and its applications has raised concern over their end-of-life management. High performance organic aerogels are typically composed of highly covalently cross-linked polymer networks, featuring pronounced chemical stability.^37^ While this robust design provides organic aerogels with exceptional material properties, it makes them virtually nonrecyclable, hindering their end-of-life management. When these aerogels reach the end of their service life cycle, they are either incinerated or disposed in landfills, leading to a loss in resources and a burden for the environment. Additionally, the economic loss of this linear produce-use-discard value chain is substantial and becomes especially important when the starting materials have high value. Reuse of aerogels reduces the burden of manufacturing energy; however, reapplying these materials eventually follows this linear economy model. Due to the lack of effective methods for recycling and valorization of the aerogel waste, valuable resources are lost, and the production of new aerogel materials continues to rely on fossil-based feedstocks and petrochemicals.

In stark contrast, the development of fully recyclable aerogels would provide the means for a sustainable circular economy. Therefore, various approaches are being explored to improve the recyclability of aerogels, specifically aiming to achieve closed-loop recycling. Recent ones focus on design of aerogel networks based on noncovalent interactions, including hydrogen bonding,^115^ electrostatic interactions,^116,117^ and metal coordination.^118^ These noncovalent bonds can be easily broken and reformed under mild conditions, enabling the recycling and reprocessing of aerogels. However, while these reversible interactions offer a potential route to recyclable aerogels, their stability poses a significant challenge. The performance of these aerogels can degrade over time due to environmental factors such as moisture uptake or temperature fluctuations, which can weaken the noncovalent interactions and reduce the material’s overall durability, raising questions about their viability for long-term applications.

A more promising strategy is the “design for recycling” approach, which encompasses the introduction of reversible covalent linkages to fabricate organic aerogels that not only have excellent properties during their useful life but also ensure their recyclability under selected conditions (Figure 6a).^119^ The introduction of such bonds to the aerogel polymeric network facilitates the on demand depolymerization back into original monomers under energy efficient conditions. As the monomers are recovered in high purity and yields, they can immediately be reused to prepare fresh aerogels with identical properties as the original ones (Figure 6b,c).^120,121^ This approach also allows partial depolymerization into soluble oligomers that can promptly be used to prepare reformed aerogels.^122^ Another key strategy is the incorporation of moieties into the aerogel structure that can selectively be cleaved under specific conditions into value-added chemicals and building blocks (Figure 6c).^121^ The design of aerogel structures containing cleavable covalent bonds effectively addresses the environmental and economic challenges of traditional aerogels, paving the way for materials that combine excellent performance with the potential for sustainable, circular aerogel economy (Figure 6).

**Figure 6:**
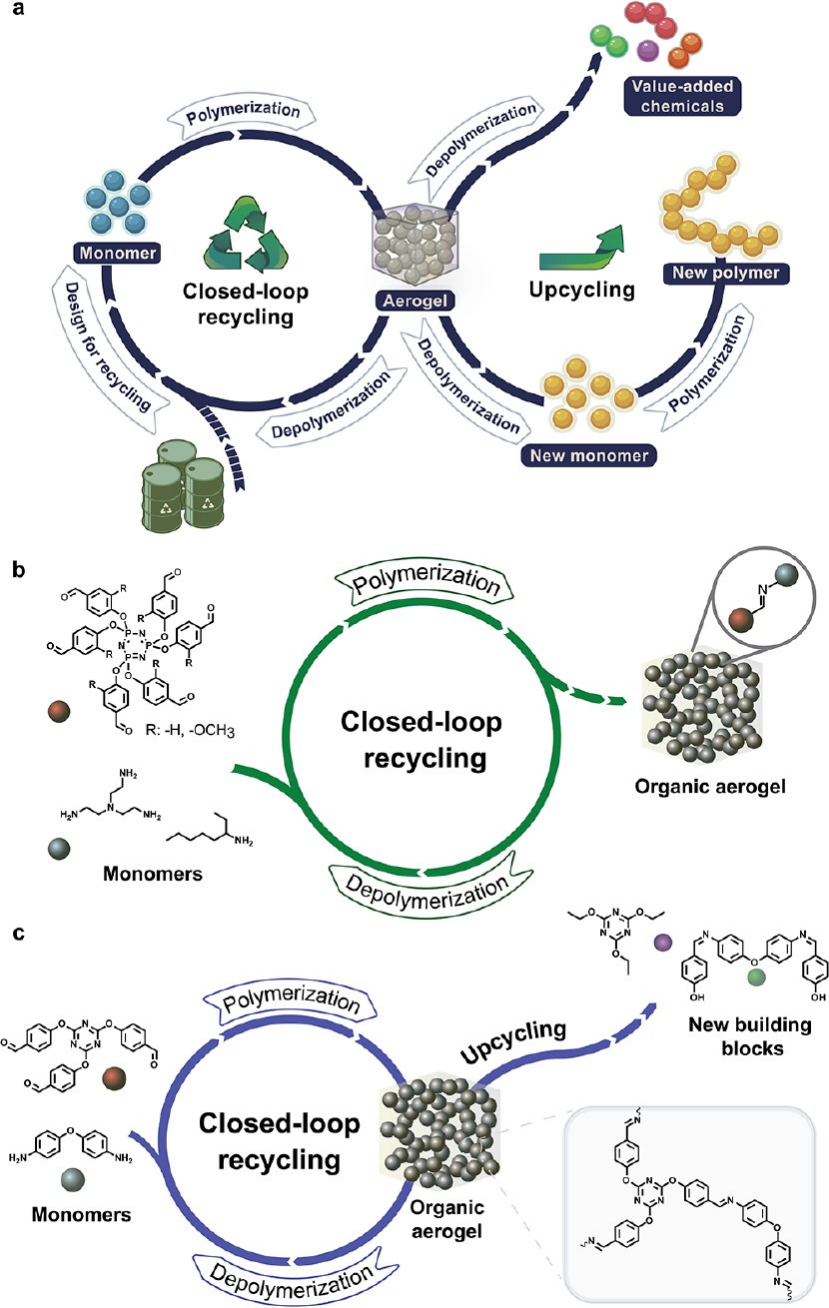
Illustration of the (a) closed-loop recycling and upcycling scheme; (b) closed-loop recycling scheme for polyimine aerogels;^120^ (c) closed-loop recycling and upcycling scheme for polyimine-cyanurate aerogels.^121^

## 6 Process Integration Strategies in Aerogel Production

This section delves into process integration strategies in aerogel production (Section 6.1), focusing on adopting green and emerging technologies to develop bespoke solutions and expand application options (Section 6.2). Sustainable, high-performance, and personalized aerogels that meet specific user requirements can be developed through these innovative approaches, while minimizing resource use and environmental impact.

### 6.1 Evaluation of Process Integration Strategies in Aerogel Production

The integration of various production processes is crucial for increasing the efficiency and sustainability of aerogel manufacturing. Key strategies include hybrid sol–gel techniques, *in situ* functionalization, and continuous flow processes.

The combination of standard sol–gel methods with advanced techniques such as supercritical drying can significantly reduce processing time and energy consumption.^123,124^ The use of supercritical CO_2_ for the drying phase in aerogel production is a highly efficient and environmentally friendly method. The supercritical drying technique not only reduces solvent residue in the final product to avoid the need for postprocessing treatments but also allows for the recycling of CO_2_, minimizing greenhouse gas emissions. Most importantly, this hybrid approach not only preserves the structural integrity of aerogels but also enhances their physical properties. The solvent exchange in gel and supercritical drying steps combined in a single apparatus can improve the efficiency of the aerogel production process.^124^ Further on, solvent management should be considered. Instead of using pure solvents, technical solvent mixtures can be applied to improve the economics of the process. However, the effect of the solvent composition on the shrinkage processes and the duration of supercritical drying should carefully be evaluated.^125^

The integration of several processes into one prompted further research on the topic of aerogel production. For instance, the processes of supercritical CO_2_ drying and sterilization were integrated into a single one for the production of aerogels suitable for biomedical applications.^126,127^ The integrated process produces decontaminated/sterile and ready-to-use aerogels while reducing processing time without compromising the aerogel’s properties for regenerative medicine purposes.

Supercritical CO_2_ can also be used for impregnation processes that enable the functionalization of aerogels, enhancing the material properties and application potential. *In situ* functionalization can be accomplished using coprecursor techniques or by incorporating functional additives into the sol–gel process.^128^ Recently, the simultaneous starch aerogel formation and curcumin impregnation were reported to enhance the curcumin’s bioavailability and storage stability.^129^ Functionalization of aerogels with natural bioactive components can also be performed after their production, for instance, via supercritical impregnation.^130^ In addition to the impregnation of neat components, bioactive compounds found in plants can be extracted and impregnated in aerogels *in situ* using the integrated process of supercritical extraction-impregnation.^131^ The resulting combination of two processes rendered savings in energy and processing time as well as minimization of extract loss and exposure to air and light. The impregnation processes can slightly change the morphology of aerogels (i.e., decrease specific surface area because of precipitation of compounds in pores), but the newly obtained functionalities of aerogels outbalance this disadvantage. Aerogels can also be used as superior carriers of hydrophobic synthetic drugs. The thus impregnated aerogels with beclomethasone dipropionate (a corticosteroid) show excellent aerodynamic properties at relevant doses, as confirmed by *in vitro* lung deposition tests, and the penetration into bronchial tissue as confirmed by *ex vivo* tests with porcine lung tissues.^132^

The shift from batch to continuous flow processes can streamline production and reduce waste and energy usage. Continuous flow reactors enable precise control over reaction parameters, leading to uniform aerogel structures and improved scalability. A continuous-mode solvent exchange system can reduce the solvent consumption during the process to one-third with respect to the batch method.^133^ In addition, the continuous supercritical drying process can efficiently produce aerogel particles.^134^ A process design involving a counter-current extraction column with freely sedimenting alginate aerogel particles was proposed using a column of 1.0 m length. The drying of aerogel particles in a shorter column (0.5 m) could be achieved by increasing the CO_2_ flow rate, resulting in a 20% reduction in the ethanol outflow mass fraction.

Finally, the integration of energy recovery systems within the production process can capture and reuse wasted heat, enhancing overall energy efficiency. The integration of industrial waste heat recovery into smart energy systems represents a main opportunity to accomplish EU climate and energy objectives.^135^ Such systems can particularly be effective in continuous flow processes where maintaining optimal reaction temperatures is critical. However, information on energy recovery system integration within the aerogel production process is still lacking.

### 6.2 Emerging Green Technologies for Aerogel Production

Emerging green technologies offer promising avenues for sustainable aerogel production. Innovative process strategies focus on the rational use of raw materials and energy, including 2D/3D-printing and plasma technology.

Aerogel materials are traditionally prepared using wet sol–gel chemistry, which involves sol preparation, sol–gel transition, post-treatment, and drying processes.^37^ Alternative methods have recently emerged, such as the solid-phase route for perovskite oxide aerogels^136^ and the gas-phase route for carbon nanotube (CNT) aerogels.^137^ These methods, whether traditional or newly developed, form the foundation for aerogel manufacturing, including both conventional and additive manufacturing techniques. Additive manufacturing, particularly 2D and 3D printing, emerged as a cost-effective production technology, suitable for both industrial scale-up and prototyping. This has positioned functional printing at the forefront of the material manufacturing revolution. Unlike traditional material removal, cutting, and assembly processes, printing is a “bottom-up” manufacturing method that builds materials from scratch. It is highly adaptable and potentially more cost-effective than traditional molding methods, allowing for the production of structurally complex parts that were previously unattainable.^138^

The first additive manufacturing of aerogels was reported in 2015, utilizing extrusion-based 3D printing of graphene oxide (GO) inks.^139^ Since then, a wide variety of aerogels, including those based on graphene,^140^ SiO_2_,^141^ resorcinol-formaldehyde (RF) polymer,^142^ polyimide,^143,144^ carbon,^145^ cellulose,^146^ metal,^147^ semiconductor,^148^ and g-C_3_N_4_,^149^ have been successfully printed. The technologies employed for printing aerogels include inkjet^150^ and microvalve drop-on-demand printing on water-repellent surfaces^151^ for microspheres, inkjet and screen for substrate-bound thin-films,^148^ and microextrusion,^152,153^ microgel-directed suspended printing,^154^ and the use of sacrificial templates^155^ for creating 3D structures.

Despite the fabrication method, aerogels are generally fragile due to their low solid content and nanoscale skeletal architecture. However, compared to traditional aerogel shaping methods, “bottom-up” additive manufacturing through functional printing offers unique advantages. In many cases, these methods allow for the design and shaping of aerogels on a microscale, addressing a significant limitation of traditional sol–gel and mechanical processing methods. The ability to control nanostructures through aerogel chemistry, combined with the macrostructural precision enabled by various printing methods, allows for adaptability and precise control over aerogel designs from nanometer to centimeter scales. This approach significantly shortens, simplifies, and improves the processes from basic chemicals or nanomaterials to functional device components, while also reducing production costs. Additionally, additive manufacturing facilitates the assembly of multimaterials with different functions and structures within different regions of an object,^144^ a capability that is typically not possible with traditional fabrication methods (Figure 7). This customization allows one to produce aerogels tailored to specific applications, minimizing material waste and enhancing performance. Recently, there has been a growing trend toward using biobased precursors in 3D printing of aerogels, such as cellulose,^156^ alginate,^157,158^ and silk fibroin,^159^ further enhancing the sustainability of aerogel production in fields like food and medical industries.

**Figure 7:**
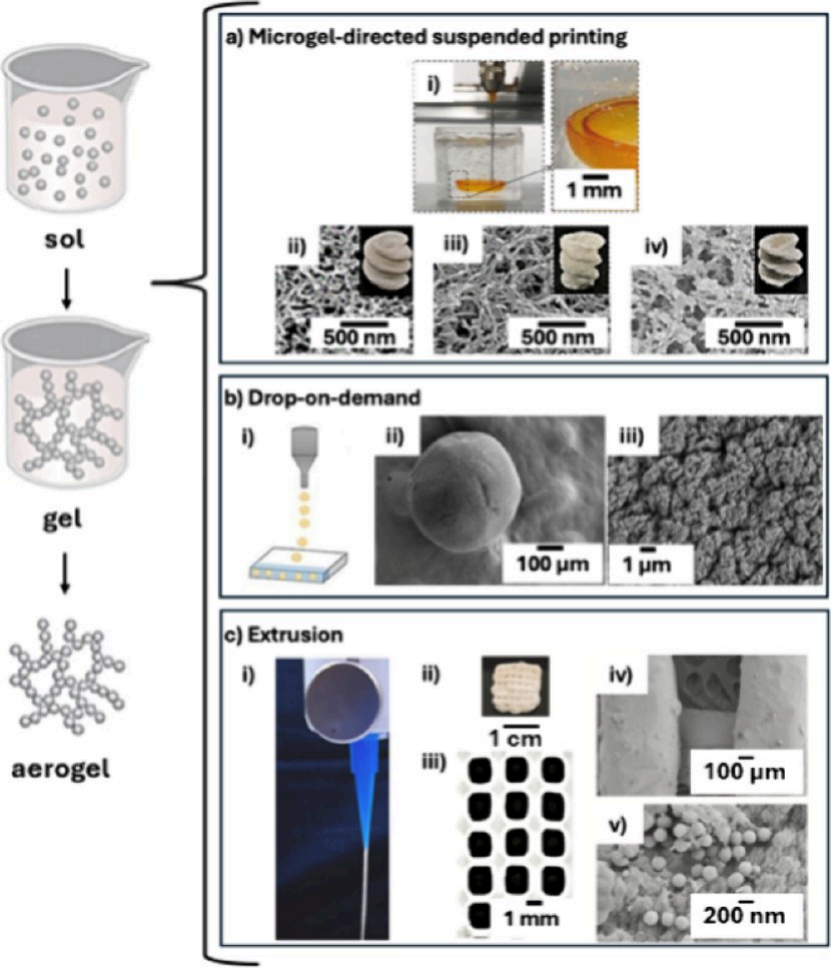
Conventional steps in the preparation of an aerogel (left). Integration of 3D-printing technology into aerogel production with selected examples (right). (a) Microgel-directed suspended printing setup: (i) printing of a Kevlar nanofiber in a microgel matrix using such method; (ii) cellulose, (iii) alginate, and (iv) chitosan aerogels (and their corresponding SEM images) obtained by using microgel-directed suspended printing.^154^ Reprinted from ref 154. Copyright 2022, American Chemical Society. (b) (i) Drop-on-demand printing process setup on a superhydrophobic surface, (ii) low and (iii) high magnifications of SEM images of antibiotic-loaded alginate aerogels microspheres printed by drop on demand.^151^ Reprinted with permission from ref 151. Copyright 2022, MDPI AG. (c) (i) Extrusion-based 3D-printing setup, (ii) visual appearance of 3D-printed alginate aerogels, (iii) 3D pattern observed on the hydrogel-based scaffolds. SEM images at (iv) low and (v) high magnifications of upconversion nanoparticle decorated alginate aerogels.^153^ Reprinted with permission from ref 153. Copyright 2024, Elsevier.

Plasma technology is another green alternative for aerogel synthesis and surface modification, producing aerogels with unique functionalities. Plasma discharge can facilitate the formation of porous structures and enhance the material surface properties without the need for harsh chemicals. Plasma treatment can be also a fast and versatile technique for deposition of protective hydrophobic and oleophobic polymer layers on hydrophilic biopolymer aerogels. For instance, hydrophobic modification of biopolymer aerogels (derived from alginate, cellulose, whey protein isolate, and potato protein isolate) was performed by cold plasma coating.^160^ While the porous structure of aerogels stayed intact during plasma treatment, polymerization inside the aerogel pores led to the generation of new porous moieties and resulted in a significant increase in the specific surface area.

## 7 Current Consideration on LCA of Organic Aerogels

LCA is the systematic mapping and evaluation of energy and material flows to critically assess the sustainability of a material or process across the entire life cycle. LCA analyzes the environmental impacts of resource consumption, material production, byproducts, waste, and emissions. LCA evaluates a specific design or process in a “cradle-to-gate” or “cradle-to-grave” approach. Obviously, such an analysis is more straightforward for materials that have been applied, or are close to being applied, at an industrial scale. For materials that are still at the laboratory or small pilot scale, many assumptions are required which limit the confidence in the analysis.^35^

According to a 2021 survey of market data,^161^ over 98% of the aerogel market is composed of silica aerogels, used predominantly for industrial, pipeline, and battery thermal insulation and/or protection. Hence, the LCA of silica aerogel production through different synthesis routes has been evaluated, albeit in different frameworks and using different methodologies.^162^ Thus, even for silica aerogels, it is therefore not possible to come up with a single measure of environmental impact. However, it is clear that, for silica aerogels, the raw materials account for a significant fraction of the embodied emissions.

The high impact of raw materials for silica aerogel production made the development of more sustainable, nontoxic, yet cost-effective raw materials a key target of the aerogel scientific community and, to some extent, the aerogel industry. As emphasized throughout this Perspective, the search for sustainability is a key driver for biobased aerogels, with commonly studied biomass raw materials including biopolymers such as cellulose, alginate, starch, chitosan, gelatin, and whey protein.^25,27,63,71^ However, no bioaerogel products are currently available on the market at industrial scale production volumes, and there are no large-scale production facilities capable of manufacturing bioaerogels in sufficient quantities to meet real-world applications. Consequently, there is an urgent need to consider the LCA of aerogels, as these materials constitute an environmental impact due to their potential production requirements, and the existing LCA studies on bioaerogel production are still limited to laboratory-scale analyses. These studies primarily focus on the “cradle-to-gate” scope, sometimes neglecting downstream processes such as utilization and end-of-life (EoL) stages (i.e., product disposal, solvent use and recycling, chemical recycling, and energy consumption).^35,163–168^

Although we are not yet able to evaluate the real industrial LCA of biopolymer aerogels, certain aspects can be predicted based on previous studies with biomass processing with other technologies, at small scale for these aerogels, or with silica aerogel in industrial production. These studies remain a critical guide for the future development of biopolymer aerogels. Although biopolymer raw materials are inherently more sustainable, extraction methods, modifications, compounding processes, and drying methods can significantly influence their overall environmental impact. As an example, corn cultivation to obtain starch causes a high marine eutrophication (MEP) (54.9%), a high terrestrial ecotoxicity (ET) (50.5%), and a high land occupation (40%).^35^ Furthermore, although most parts of the needed chemicals during the fabrication of aerogels do not remain in the end product, the way in which they are handled is critical for minimizing environmental effects, especially during the stages of solvent exchange and aging steps, where large amounts of solvents are used. Ethanol is one of the most utilized solvents, and therefore, it can represent ca. 50% of the global warming potential (GWP) in all types of aerogels and is one of the main contributors to abiotic depletion potential (ADP) and photochemical oxidation.^169^ Methanol is also commonly used, and by reducing the number of solvent exchanges, carbon emissions can be reduced by up to 7-fold,^170^ although this methanol presents serious toxicological impacts on health and environment when compared to ethanol, which is an aspect that can not be disregarded. The drying process, in particular, remains a major challenge for LCA evaluations of aerogels because its significant energy and solvent demands strongly depend on the specific implementation, e.g., on the detailed equipment and process engineering. LCA of the drying step in commercial silica aerogel production provides insights for future optimizations in other aerogel sources. The drying process is both a challenge and a defining feature for aerogels, as it significantly influences their properties and environmental impact. Different drying methods have varying environmental impacts. Freeze-drying, commonly used for biopolymer aerogels, is known for its very high energy consumption.^162,168,171^ Ambient pressure drying and supercritical drying involve substantial solvent use due to the close correlation between drying processes and solvent types, leading to both high energy consumption and environmental concerns.^172,173^ Recent studies have highlighted the need for enhancing the sustainability of the production through processes that minimize, eliminate, or recycle solvents and CO_2_ (ca. 95% recycling rates can be obtained) without compromising aerogel properties.^35,166,174^ Furthermore, optimizing energy consumption in fabrication processes and utilizing renewable energy sources can greatly reduce the carbon footprint.

The environmental repercussions of aerogels can be evaluated through six parameters: GWP, acidification potential (AP), ADP, eutrophication potential (EP), ozone depletion potential (ODP), and photochemical ozone creation potential (POCP).^175^ The transition from lab-scale aerogels to pilot and industrial scales would reduce in a big proportion the environmental impacts. For example, in the scale-up production of starch aerogels from lab-scale to pilot, the GWP and AP reductions would be 72%, while from lab to industrial, they would be 95%.^166^ The reductions in EP would be 61% and 93% for pilot and industrial, respectively, and in ODP, 81% and 96%, respectively; the electricity use would be reduced to 89% and 99% and the primary energy demand (PED), to 74% and 95%.

Aerogel waste management introduces further uncertainties in LCA. Biopolymer aerogels, primarily composed of polysaccharides or proteins, are generally considered nontoxic and biodegradable. However, their disposal can be complicated by the inclusion of inorganic or organic cross-linking agents (e.g., chitosan) or surface modifications (e.g., hydrophobization). Composite structures further add to this complexity, and there is currently a lack of comprehensive information regarding their disposal methods and long-term environmental impacts. Finally, tailored LCAs are required for different application scenarios, from thermal insulation to environmental remediation and biomedicine, each with distinct environmental impacts. Addressing these challenges will be crucial for advancing biopolymer aerogels toward sustainable large-scale production and real-world applications.

## 8 Conclusions, Future Directions, and Final Remarks

Despite the efforts to revalue waste materials toward innovative uses, aerogels produced directly from waste are still in their early stages. It is important that circularity not only focuses on waste recycling and reuse but also considers that the processes for aerogel manufacturing or postmodification must be sustainable and scalable, with products being ideally reusable or recyclable. LCA for the production at all levels (from waste collection to end-of-life disposal/reuse of the developed materials) must be done by an economic analysis of the environmental impact considering the entire process.^176^ Economic viability cannot be ensured solely by defining aerogels as high-value materials, but specific indicators (e.g., financial rate of return to companies and society, benefit-cost ratio) should be evaluated. Also, the alignment with specific United Nations SDGs should be reviewed.^177^ In summary, there is still a research gap on the sustainable production of aerogels, which will motivate researchers to develop future aerogel-based materials.

Aerogels based on natural resources/waste fractions have some common challenges related to the large variations in the quality and composition of the raw materials. Even when the same biomass is used, this does not guarantee consistent biomass composition, as it is susceptible to significant variations depending on various factors, such as climate history. Consequently, the properties of the fractions and final products (e.g., color, mechanical and chemical stability) may vary, potentially affecting the process. As an example, impurities originating from plant proteins may lead to catalyst deactivation during initial biomass processing.^178^ Consequently, the aerogel production process must be continuously adjusted to accommodate the varying raw material quality, which is currently not considered in process analysis and LCA. In order to tackle this problem, stronger involvement of high-resolution climate modeling in the planning of material production processes is required. Recently, the term “climate-informed engineering” was suggested to address this issue^179^ by training a new generation of engineers, who consider climate information in their engineering services similar to the way economic aspects are considered in their products. Given that biobased aerogel production combines numerous aspects of material science, engineering, and chemistry relevant all over the world, it can serve as a demonstration field for this timely initiative and thus also improve the process sustainability.

On the other hand, the evaluation of the human and environmental impact will help to advance in the development and improvement of aerogels to address new health and environmental challenges within the context of the circular economy while complying with the *One Health* concept established by the World Health Organization.^180^ The ecotoxicity and health risk assessment of aerogels are not specifically regulated, as aerogels do not require registration as nanoforms. Nevertheless, their nanostructured design can raise concerns about a possible hazard assessment, which needs to be addressed. For regulatory purposes, several aspects should be considered. As the toxicity inherent to aerogel exposure is not expected in general, the bioactivity of inhalable or ingestible fragments due to their high inner surface area can raise concerns.^181^ The pulmonary route due to inhalation of aerogel nanoparticles as material dust and the consequent pulmonary deposition^182^ is the main route of human exposure to the eventual toxicity of aerogels, since fine particles with diameters smaller than 2.5 μm penetrate the alveoli and even reach the cardiovascular system when smaller than 0.1 μm. Professional exposure during the installation and removal activities of insulation materials is one of the most frequent applications where humans are exposed. The use of aerogels on an industrial scale requires the implementation of safety regulations for workers involved in their production and exposure to these nanostructures. Moreover, these particles can be dispersed into the surrounding environment and can circulate in air, soil, and water. Thus, global regulation of their ecotoxicity is needed to prevent any risks to the health of the biosphere and to limit their associated pollution.^183,184^ Additionally, appropriate safety regulations are involved in the manufacturing of aerogel products, identifying hazards for the environment and human and animal health.^185,186^ Since aerogels can comprise several chemical compounds, the composition of each ingredient should be classified as “nonhazardous” in order for the aerogel itself to be considered nonhazardous.^187^

There are still insufficient studies on the potential toxicity of certain aerogels. Recently, a systematic toxicological workflow, used to test nanomaterials, was proposed to evaluate and classify aerogels based on their safety profile.^182^ Nineteen aerogels, both organic and inorganic, were compared using a 3 Tier evaluation. The materials’ biosolubility and oxidative potential were tested in Tier 1, the material’s toxicity in alveolar macrophages *in vitro*, in Tier 2, and intratracheal instillation in Wistar rats, in Tier 3. All aerogels showed good biocompatibility, except for the case of polyurethane aerogels where a low toxicity potential was detected in Tier 2. From all tested aerogels, only moderate, transient, and reversible inflammation in the lung was found for polyurethane aerogels in Tier 3. In addition to these basic toxicity tests, it is mandatory to evaluate long-term exposition, repeated administration evaluation, bioabsorption, distribution, metabolism, and excretion to ensure safe use in biomedical applications.^188,189^

From a data mining perspective, leveraging AI and machine learning algorithms can optimize production parameters, predict process outcomes, and reduce resource consumption. AI-driven models can identify the most efficient pathways for aerogel synthesis, minimizing the use of raw materials and energy. This direction has been successfully used for aerogel production and application optimization. For instance, it was shown that deep reinforcement learning (DRL) with the diffusion-limited cluster–cluster aggregation (DLCA) algorithm can be applied for microstructural optimization of silica aerogels.^190^ Machine learning-based multiobjective optimization using the NSGA-II-study of modeling has been applied for an aerogel glazing system in the subtropical climate in order to minimize the total heat gain and maximize the indoor illuminance transmitted through the system.^191^ Moreover, an artificial neural network (ANN) was developed for predicting the fractal properties of silica aerogels, given the input parameters for a DLCA algorithm. This approach of machine learning replaces the necessity of first generating the DLCA structures and then simulating and characterizing their fractal properties.^191,192^ Collaborative robotics were also tested in combination with machine learning tools to accelerate the design of conductive aerogels from mixtures of Ti_3_C_2_T_*x*_ MXene, cellulose, and gelatin with programmable properties.^193^

The visibility of aerogel-based materials and their impact as a top emerging technology have dramatically increased in the past decade. Overall, organic aerogels are multifunctional materials that can be obtained from synthetic or natural materials and have a wide range of applications. The industrial sectors for waste collection (food, textiles, cosmetics, cattle, demolition materials), biorefinery (lignocellulose production), and applications (construction, biomedicine, pollution remediation, critical raw materials recovery, food) are the target groups for expanding the sustainable production and use of aerogels. The awareness and possibilities of these advanced materials can be boosted by new and ongoing international initiatives among the aerogel scientific community. These initiatives include the implementation of an international association on aerogels looking for a harmonized voice for the scientific community, the general public, and other stakeholders and to increase networking, training, and other outreach activities. A redefinition of the aerogel term by the IUPAC Association is urgent and has been recently deployed to meet a consensus aiming at limiting the array of materials falling within this material category.

The snapshot on aerogel technology provided in this Perspective unveils a promising present and near-future market outlook alongside an active and growing scientific community. The glimpse of mid-to-long-term future trends on aerogel technology and applications herein provided is optimistic with novel uses and fast-growing market shares. It also highlights prominent research to adapt conventional aerogel production toward the paradigms of circular economy and raw material and energy efficiencies. The sustainability principle must be definitely tackled within the aerogel community. Venues for progress and current gaps to be filled in aerogel technology are identified, and intense research efforts are needed from different approaches and domains. There are already incipient international collaborative efforts on research, training,^29,194^ and events specifically focused on the cross-fertilization of ideas on this environmental aspect for aerogels, as well as innovation grants funded by public funding bodies^195,196^ to boost business plan initiatives with efficient communication between innovators, industrial players, and business developers.
